# Atypical Plasmacytic Proliferation in a Case of C3 Glomerulopathy: Pathophysiology Demystified

**DOI:** 10.1177/2324709617690746

**Published:** 2017-02-01

**Authors:** Osama Elfituri, Nathan Aardsma, Suman Setty, Frederick Behm, Kimberly Czech

**Affiliations:** 1 Department of Pathology, University of Illinois, Chicago, IL, USA; 2 Department of Pediatrics, University of Illinois, Chicago, IL, USA

**Keywords:** kidney, monoclonal, C3 glomerulopathy

## Abstract

An 11-year-old Hispanic female underwent evaluation of asymptomatic proteinuria and hematuria. The patient denied fever, edema, and gross hematuria. Urinalysis showed mild proteinuria, and a urine microscopic examination revealed red blood cells. Screening tests for glomerulonephritis revealed a low C3 and negative ANA, ASO, DNAse-B, and ANCA. Histological examination of a renal biopsy specimen showed glomeruli with endocapillary proliferation, a predominant C3 deposition in the capillary loops by immunofluorescence, and electron dense deposits in the mesangium, paramesangium, and capillary walls by electron microscopy consistent with a diagnosis of C3 glomerulopathy. An interstitial plasmacytosis was also present with focal clustering of plasma cells, which were found to be kappa light chain restricted by in situ hybridization suggestive of a clonal proliferation. One can speculate that these plasma cells may be directly responsible for the renal pathology that was seen.

## Case Report

**Figure 1.**
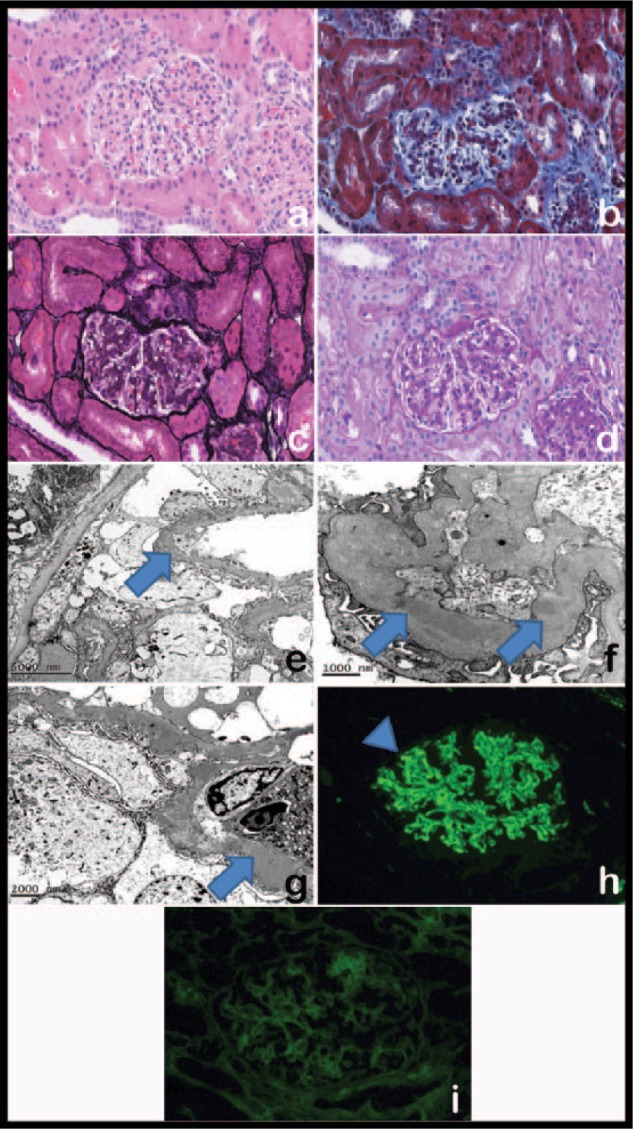
Representative kidney biopsy findings. Kidney biopsy revealed (a-d) a membranoproliferative pattern of injury on light microscopy (hematoxylin and eosin, Masson trichrome, Jones’ silver, and periodic acid Schiff stain, respectively, original magnification 40×) and (e-g) intramembranous, subepithelial, and subendothelial deposits on electron microscopy (arrows) (original magnification 5000×, 40 000×, and 25 000×, respectively). (h) Immunofluorescence microscopy with antibody to C3 demonstrates diffuse, global glomerular basement membrane and mesangial positivity in a granular pattern (head arrow) (original magnification 200×). (i) Negative reactivity with IgG (original magnification 200×).

An asymptomatic 11-year-old Hispanic female was referred to a pediatric nephrologist and found to have proteinuria and hematuria on screening urine analysis. The patient was growing and developing normally and denied fever, edema, joint pain, headache, dizziness, dysuria, and gross hematuria. She was not hypertensive with a manual blood pressure 109/68 (95th percentile is 121/79). Urinalysis showed mild proteinuria with a urine protein to creatinine ratio of 0.92 mg/dL (first morning void 0.38 mg/dL) and 24 hour of 0.624 g/24 hours. Urine microscopic examination revealed 21 red blood cells per high-power field and no casts were reported. A repeat urinalysis showed persistent hematuria and mild proteinuria over the course of 6 weeks, which prompted screening tests for glomerulonephritis. Due to the presence of persistent hematuria (17-21 red blood cells per high-power field), serology was sent for C3/C4, which revealed a low C3 at 11 mg/dL (normal C3 >76 mg/dL) and normal C4 at 15 mg/dL. ANA, ASO, DNAse-B, and ANCA serologies were negative. C3 nephritic factor was within the normal ratio of 0.17 (normal range 0.00-0.30), factor I level 29.7 µg/mL (normal range 29.3-58.5 µg/mL), factor H (B1H) level 191 µg/mL (normal range 160-412 µg/mL), and normal serum protein electrophoresis pattern. Urine protein electrophoresis was done to reveal 64.9% albumin, 10.4% alpha-1, 9.9% alpha-2, 12.8% beta globin, and 2.0% gamma globin. No monoclonal spikes and paraprotein was detected. An ultrasound of the abdomen showed mildly increased renal cortical echogenicity with no evidence of obstruction. A month later an ultrasound-guided core percutaneous renal biopsy was performed and a total of 84 glomeruli were examined. As shown in Figure 1a, microscopic examination by hematoxylin and eosin staining revealed that the majority of glomeruli had global endocapillary proliferation. The Jones’ silver stain revealed “vacuolation” of the basement membranes suggestive of deposition of material within the basement membranes. No glomerular capillary loop fibrin or crescents were identified. There was a minimal tubular atrophy. Muscular arteries were unremarkable. Prominent nodular arteriolar hyalinosis was present. Vascular thrombi, fibrointimal hyperplasia, and vascular recanalization were absent.

There were focal dense interstitial aggregates of plasma cells mixed with occasional lymphocytes and eosinophils. The plasma cells were found to be positive for kappa light chains by in situ hybridization using DNA probes. Rare glomerular and tubular eosinophils were also identified. Masson trichrome and Jones’ silver special stains were also performed and supported these findings as shown in Figure 1b and c, respectively.

Electron microscopy revealed glomeruli with mainly effaced foot processes (Figure 1e, f, and g). The glomeruli had marked wrinkling of basement membranes with electron densities in the mesangium, paramesangium, and on the subendothelial and subepithelial surfaces and intramembranous location of the capillary lumen. Some large “sausage-like” were present along the subepithelial surface of the basal lamina. Tubules were unremarkable.

Immunofluorescence microscopy was positive for a predominant C3 deposition in the mesangium and glomerular capillary walls, granular, and moderate in intensity with focal deposition of C3 seen in the wall of blood vessels (Figure 1h). Immunofluorescent reactivity was negative for immunoglobulins IgG, IgM, and IgA (Figure 1i).

In situ hybridization for kappa and lambda light chains using DNA probes was performed and showed close to 100% of plasma cell nuclei positive for kappa light chains and only 1% for lambda light chains. A diagnosis of C3 glomerulopathy was considered with monoclonal kappa chains plasma cell infiltrates.

## Discussion

The patient is a young 11-year-old female presents with no complaints or symptoms found to have microscopic hematuria on screening urinalysis for a school physical. Workup at an outside hospital and referral testing at our institution yielded mild proteinuria and hematuria, which persisted for 6 weeks. A renal biopsy demonstrated the presence of a predominantly glomerular deposition of C3 fraction of complement in the mesangium, paramesangium, and glomerular capillary wall associated with a small clonal proliferation of plasma cells. A review of the literature revealed that this association has been observed in older individuals having bone marrow or serum evidence of a plasma cell dyscrasia.^1,2^ Although this patient did not have a detectable clonal process in the serum or urine, the renal biopsy findings of monoclonal plasma cells and C3 deposition are intriguing. Also, the presence of glomerular C3 and lack of immunoglobulins qualifies the process as a C3 glomerulopathy.

C3 glomerulopathy is characterized by glomerular deposition of the C3 complement fragment as a result of dysregulation of the alternative pathway in the complement cascade.^3,4^ Recently, the location, type, and ultrastructure appearance of the deposit have been described and these features are necessary for a diagnosis of C3 glomerulopathy (C3G). Membranoproliferative glomerulonephritis (MPGN), types I, II, and III, have been included in this entity.^4-6^ The most common form of C3G is MPGN I with predominantly subendothelial deposits, followed by MPGN II, also known as dense deposit disease (DDD).^3,7^ Patients with DDD have circulating autoantibodies known as C3 nephritic factor. C3 nephritic factor is determined as a ratio of C3 fragments to intact C3. The presence of C3 nephritic factor stabilizes the C3 converting enzyme, resulting in increased breakdown of C3 and in a higher ratio. However, C3 nephritic factor can also be detected in individuals without glomerular disease.^8^ MPGN III has both subepithelial and subendothelial deposits. In our patient, the C3 nephritic factor ratio was 0.17, which is within the normal range (the normal range is 0.00-0.30). Also, postinfectious glomerulonephritis (PIGN) may present with a similar histologic picture.^9^

Given the heterogeneity of diseases encompassed by the term C3GN, a definitive diagnosis and determination of etiology should be based on a combination of light microscopy, electron and immunofluorescence microscopy, serologic tests, and clinical features.

In summary of the case being presented, the persistent proteinuria and hematuria was accompanied by biopsy findings of glomerular disease. Examination of renal tissue by light, immunofluorescence, and electron microscopy revealed the presence of a dominant C3 deposition in the glomeruli. Although this picture can be seen in both PIGN and C3 glomerulopathy^9^ and PIGN can have a similar clinical presentation, the absence of ASO and DNase-B antibodies and absence of clinical stigmata excluded the possibility of PIGN.

Activation of complement in the classical pathway with cleavage of C4 and subsequent binding of the C4d fraction covalently to the site of activation is commonly observed in the antibody-mediated rejection pathway posttransplantation. Also, Sethi and colleagues^10^ and Cook^11^ proposed the use of C4d immunohistochemical staining to differentiate PIGN and C3 glomerulopathy, where C4d would be absent in C3 glomerulopathy. Immunohistochemistry for C4d was negative in the present case.

Although the primary cause of C3 glomerulopathy, particularly MPGN, is a de novo process, secondary insults such as monoclonal gammopathy can produce similar features. This process is believed to be driven via the dysregulation of alternative complement pathway and consequently, glomerular deposition of monoclonal immunoglobulins or C3 immune complexes, resulting in C3 glomerulopathy.^1,2,7,9,12,13^

A consensus report placed both C3 glomerulopathy and PIGN under the umbrella of “glomerulonephritis with dominant C3” for cases with a dominant staining for C3.^3^ It is notable in this case that serial serum C3 levels were low values from undetectable to less than 20 despite treatment. Renal biopsy tissue was processed for immunofluorescence staining was positive for a dominant glomerular C3 deposition in combination with the monoclonal kappa chains restricted plasma cell infiltrates. This coexistence indicates that the monoclonal immunoglobulins may indeed be the cause of the C3 deposition.

Based on the presence of only microscopic hematuria with minimal proteinuria, she was started on an angiotensin-converting enzyme inhibitor with questionable compliance/adherence to medication. Six months after initial diagnosis and treatment with an angiotensin-converting enzyme inhibitor, she developed worsening hematuria and nephrotic syndrome with urine protein to creatinine ratio of >5.9. She was placed on high-dose oral steroids with minimal response in terms of remission of both nephrotic syndrome and correction of low C3. Her renal disease continues to progress and she is undergoing additional treatment options.

A clinical study performed by Sethi and Rajkumar at Mayo clinic revealed that in plasma cell disorders, the presence of monoclonal immunoglobulins causes a dysregulation of the alternative pathway resulting in mesangial and capillary injury. This leads to glomerular deposits of complements without significant immunoglobulin deposits, thus leading to an immunoglobulin-negative C3 positive glomerulonephritis (C3 glomerulopathy).^10^

In addition, the 2008 World Health Organization classification of plasma cell neoplasms describes a disorder with monoclonal deposits in the glomerular basement membrane termed monoclonal immunoglobulin deposition disease, which is characterized by presence of nonfibrillar monoclonal deposits and can be classified by the type of immunoglobulin chain present in the deposits (light chain, heavy chain, or a combination of light and heavy chains).^14,15^ In the current case there were no glomerular immunoglobulin deposits. Moreover, not all cases of plasma cell dyscrasia have clinical or laboratory features of the disease, and these have been termed smoldering and nonsecretory myeloma, respectively.^14^

## Conclusion

We conjecture that we have encountered an early clonal plasma cell process in the kidney preliminary to the development of systemic involvement. This biopsy provides insights into the process of development of proliferative glomerulonephritis and suggests that an undetected monoclonal gammopathy may be the basis of proliferative glomerulonephritis in more cases that has been previously thought to occur.
